# Habitat Selection and Heterospecific Flocking Associations of a Single Male Scaly-Sided Merganser (*Mergus squamatus*) over Three Winter Seasons in Northern China

**DOI:** 10.3390/ani16162579

**Published:** 2026-08-18

**Authors:** Yongbin Zhao, Yanan Hao, Bing Liu, Guodong Yi, Jun Liu, Zhigao Liu

**Affiliations:** 1 College of Life Sciences, Jilin Normal University, Siping 136000, China; syding@jlnu.edu.cn (Y.Z.);; 2 College of Educational Sciences, Jilin Normal University, Siping 136000, China; 3 Forestry Pest Control and Quarantine Wildlife Protection Office of Liaoning Benxi Manchu Autonomous County, Benxi 117100, China

**Keywords:** *Mergus squamatus*, mixed-species flocking, habitat selection, ice-free winter habitat, human disturbance

## Abstract

The Scaly-sided Merganser (*Mergus squamatus*) is an endangered duck endemic to East Asia. It usually gathers in single-species flocks through winter. Existing field records focus almost entirely on southern winter flocks. No prior work has described lone individuals spending winter in northern China. We conducted standardized three-year winter surveys (2019–2022) across five isolated ice-free river patches along the Taizi River, Liaoning Province, northern China, to document habitat use and flocking behavior of one solitary male. Despite the availability of five discrete overwintering sites, the focal bird only occupied two patches characterized by dense riparian concealment and low human disturbance, avoiding open, highly disturbed sites. Resource Selection Function (RSF) modeling indicated habitat attributes (concealment and human disturbance) strongly predicted site use (AICc weight = 0.79), whereas the abundance of heterospecific flocking partners (Common Mergansers *Mergus merganser*) exerted negligible influence (AICc weight < 0.01). This solitary male associated only with Common Mergansers, the species with the closest phylogenetic relatedness (mitochondrial genetic distance = 0.04365), and never aggregated with other sympatric waterbirds. Model comparison confirmed phylogenetic relatedness outperformed dietary similarity as a predictor of heterospecific flocking (AICc weight = 0.76). Our three-year observations of this single individual demonstrate that, when conspecifics are absent and suitable winter habitat is limited, this male prioritized high-quality, low-disturbance ice-free patches over social aggregation opportunities, and selectively formed mixed flocks with its closest phylogenetic congener. This multi-year case study provides rare empirical baseline data for understanding winter behavioral trade-offs of endangered cavity-nesting waterfowl under severe frozen river conditions, though all conclusions herein apply only to this focal male and cannot be generalized to the entire species.

## Simple Summary

The Scaly-sided Merganser (*Mergus squamatus*) is an endangered East Asian waterfowl. It typically winters in large monospecific flocks across southern China. By contrast, lone overwintering birds are extremely rare in northern frozen river landscapes. Over three winters (2019–2022), we tracked a solitary male Scaly-sided Merganser in five ice-free river patches in Liaoning Province to assess its habitat use and mixed-species flocking behavior. Our habitat models showed that the focal bird strongly preferred sheltered, ice-free reaches with little human disturbance. This preference was stronger than its tendency to associate with Common Mergansers (*Mergus merganser*), suggesting that habitat quality takes priority over flock availability when selecting a winter site. The exclusive association with Common Mergansers, meanwhile, appears to reflect phylogenetic relatedness rather than diet—these two species are close genetic relatives, with similar diving behavior and anti-predator strategies. Other fish-eating waterbirds, which used more open and heavily disturbed habitats, were never observed flocking with our focal bird. This case study highlights the conservation importance of small, undisturbed riparian ice-free refugia for isolated endangered mergansers under harsh northern winter freezing conditions. All behavioral patterns observed apply only to this single male, and further large-scale watershed surveys are needed to verify whether these rules hold for other solitary conspecifics.

## 1. Introduction

For birds, securing adequate food resources, avoiding predation, and achieving successful reproduction are fundamental requirements for survival and population persistence [1]. Over evolutionary time, birds have developed the ability to select suitable habitats across landscape, local, and microhabitat scales [2]. They have also evolved both intraspecific and interspecific flocking, which enhances foraging efficiency and reduces predation risk [3]. However, the strict habitat specialization that enables birds to thrive in specific environments can also increase their vulnerability under rapid natural or anthropogenic environmental change [4]. Similarly, flocking entails costs, including intensified resource competition and elevated parasite transmission risk [5]. Understanding how rare or specialized bird species balance refined habitat selection with the costs and benefits of interspecific flocking remains an important unresolved question, particularly for taxa that rarely form mixed-species flocks.

The scaly-sided merganser (*Mergus squamatus*) is an endangered waterfowl endemic to East Asia and is currently listed as Endangered (EN) on the IUCN Red List of Threatened Species (BirdLife International, 2025) [6]. It breeds across a restricted zone spanning the southeastern Russian Far East, Northeast China, and northern North Korea, while its primary wintering range lies within the Yangtze River basin in southern China [7]. Previous studies have indicated that this species predominantly forms intraspecific flocks during the non-breeding season and exhibits high vigilance [8]. Previous studies have indicated that this species predominantly forms intraspecific flocks during the non-breeding season and exhibits high vigilance [8]. To date, no published study has documented mixed-species flocking in the Scaly-sided Merganser. However, our volunteer collaborator (see Acknowledgments) have observed wintering individuals in southern China occasionally tolerating other waterbirds nearby, including Little Grebe (*Tachybaptus ruficollis*) and Mandarin duck (*Aix galericulata*). Coordinated behaviours, such as synchronized foraging or collective vigilance, were never observed. This suggests these aggregations are passive assemblages rather than functional mixed-species flocks. To date, few studies have investigated how this merganser balances habitat selection and social grouping choices. Extreme habitat limitation and the absence of conspecifics may unpack these behavioral decision-making strategies.

In northern China, most water bodies freeze over in winter. However, certain reaches downstream of hydropower stations and hot spring inflows remain ice-free, creating critical winter refugia for waterbirds [9]. This region is not a typical wintering ground for the Scaly-sided Merganser. Nevertheless, a small number of individuals may fail to complete southward migration due to navigational errors, juvenile dispersal, injury, or extreme weather events, and thus remain in the north. From 2019–2022, we conducted a three-year winter survey across five ice-free reaches of the Taizi River basin in Benxi, Liaoning Province, China. We consistently recorded a solitary Scaly-sided Merganser overwintering in this area. This individual associated with Common Mergansers (*Mergus merganser*), and its behavioral rhythms synchronized with theirs. This pattern differs from that of conspecifics wintering in southern China, as previously reported [10].

We addressed two individual-level behavioral questions arising from this unique three-year observation: (1) When suitable ice-free habitat is limited and conspecifics are absent, does this solitary male prioritize habitat quality or heterospecific flocking opportunities? (2) What trait drives its selective association with only Common Mergansers among all sympatric waterbirds? We tested two hypotheses: first, concealment and low anthropogenic disturbance would be stronger predictors of site use than the presence of potential flocking partners; second, phylogenetic relatedness would better explain heterospecific association patterns than shared dietary guilds. To address these questions, we employed a Resource Selection Function (RSF) framework, a standard approach for quantifying habitat preference by comparing used versus available sites [11]. This approach allows us to determine whether habitat attributes or the presence of potential flocking partners better predicts site use. Additionally, we examined the potential role of heterospecific interactions with Common Mergansers, as mixed-species associations in waterfowl can provide anti-predator and foraging benefits in other systems [3].

## 2. Methods

### 2.1. Study Area

**Figure 1:**
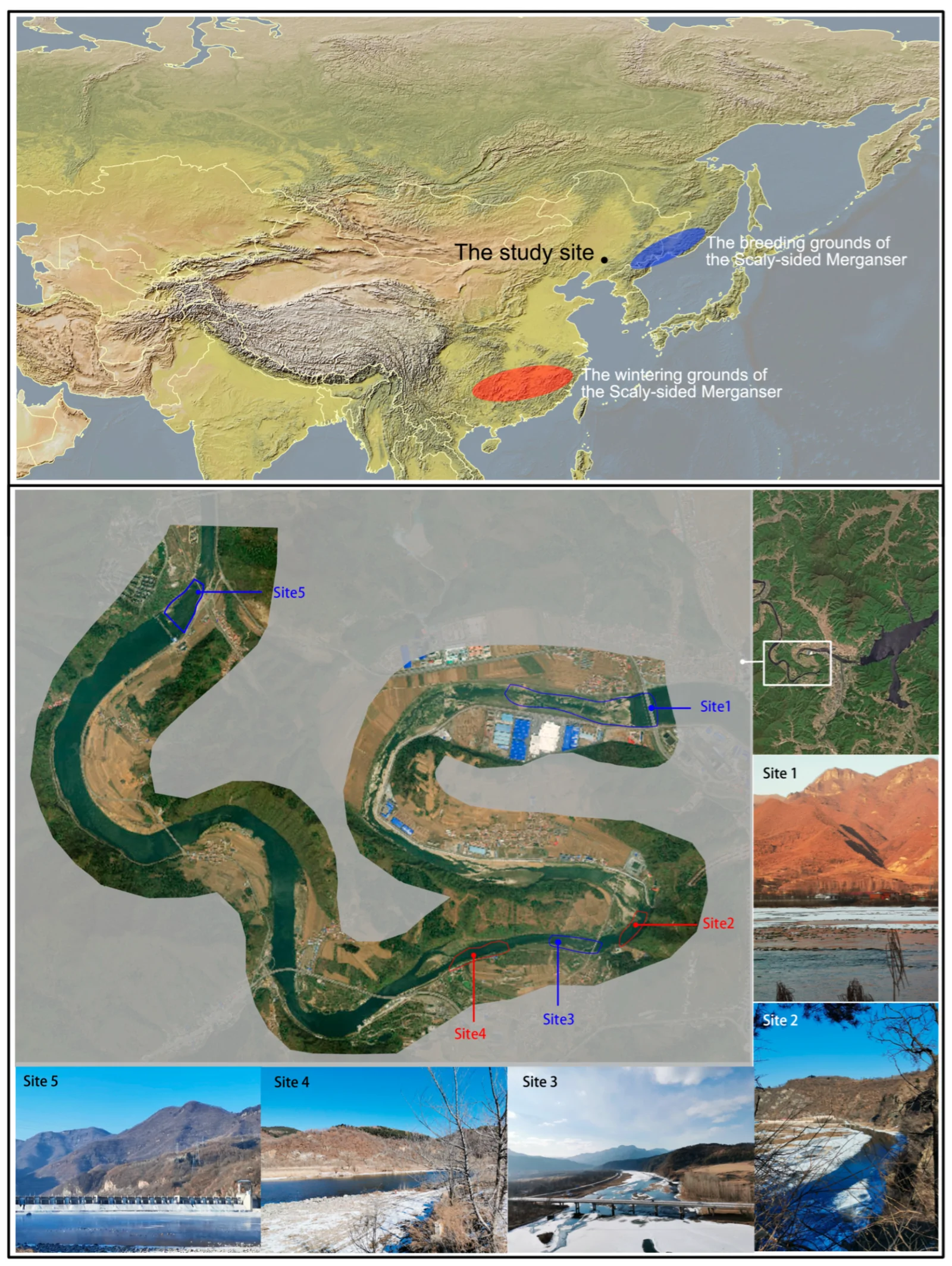
Overview of the study area and sampling-site distribution for winter-habitat observation of the Scaly-sided Merganser. (**Top**) The regional-scale map displays the species’ breeding grounds (blue shaded zone) and wintering grounds (red shaded zone), with the black dot marking the location of our river-reach study site. (**Bottom-left**) Satellite imagery of the meandering river channel showing five unfrozen sampling patches (Site 1–5). Red labels indicate sites where the merganser was recorded, and blue labels represent sites without merganser occurrence. (**Bottom-right** and **bottom**) Field photographs showing the on-site environment of each sampling location. National-boundary shape-file data were obtained from Natural-Earth (1:110 m) [13]. Satellite-image base-map sourced from Tianditu [14]. Map plotted in ArcGIS 10.8.1.

The study was conducted along the Taizi River in Benxi Manchu Autonomous County, Liaoning Province, northeastern China (approximately 41° N, 123° E). The region has a temperate continental climate, with winter temperatures ranging from −25 °C to 3 °C (December to February) [12]. Five distinct ice-free river patches were distributed across the study area, maintained by continuous warm water discharge from hot springs and hydropower facilities. Covering surface areas of 1000 to 10,000 m^2^, these ice-free patches were isolated by frozen river segments, creating independent habitat units for overwintering waterfowl (Figure 1). 

### 2.2. Field Surveys

From December to February 2019–2022, each ice-free patch was surveyed 10 times per month between 8:00–17:00. We used 12× binoculars (Swarovski Optik, Absam, Tyrol, Austria) to scan the water surface. Each survey session lasted 9 h per day (8:00–17:00). When the Scaly-sided Merganser was detected, we documented its behaviour via instantaneous scan sampling at fixed 5-min intervals following Martin and Bateson [15]. Identification as an adult male Scaly-sided Merganser was confirmed by the presence of a slender crest, a long red conical bill, bright black upperparts, white lower back and rump, and black scalloped patterns along the flanks. Females and juveniles were ruled out based on the lack of a brownish head, bluish-grey upperparts, and an indistinct crest [16]. For all sites, we recorded waterbird community composition, habitat characteristics, and human disturbance. Waterbird surveys included species identification and abundance estimation, with direct counts for small groups and unified group counting for large flocks. Habitat variables included water width (m, laser rangefinder), and flow velocity (slow <0.3 m/s, moderate 0.3–0.5 m/s, fast >0.5 m/s); we also quantified concealment (1 = open, 2 = partial, 3 = fully sheltered). Human disturbance indicators comprised distance to nearest road, hourly human activity frequency (1 = no activity, 2 = occasional, 3 = frequent), and ambient noise level (1 = quiet, 2 = moderate, 3 = loud). The five sites exhibited marked variation in habitat characteristics (Table 1). Sites 2 and 4 were characterized by high concealment, low human disturbance, and slow to moderate flow velocities, whereas sites 1, 3, and 5 were open with direct exposure to roads, frequent vehicle traffic, and (for site 3) fast-flowing water. Spot measurements were taken with a digital thermometer in December 2020, while continuous 60-day automatic recording was conducted via electronic loggers at three-minute intervals. Throughout the entire survey period, water temperatures across all sites remained stable, ranging from 1 to 4 °C.

### 2.3. Genetic Distance and Diet

To compare phylogenetic relationships, we retrieved complete mitochondrial DNA sequences of the eight waterbird species recorded at our five survey sites (*Anas platyrhynchos*, *Anas zonorhyncha*, *Podiceps cristatus*, *Mergus squamatus*, *Tachybaptus ruficollis*, *Tadorna ferruginea*, *Mergus merganser* and *Mergellus albellus*) from GenBank (accession numbers: EU009397.1, MZ593724.1, NC_008140.1, NC_016723.1, NC_024594.1, NC_024640.1, NC_040986.1 and OZ310727.1) [17] and calculated genetic distances between the Scaly-sided Merganser and each sympatric species using MEGA12 version 12.1 [18]. Pairwise distances were estimated using the Maximum Composite Likelihood model with 1000 bootstrap replicates, following standard phylogenetic practice. Dietary information was compiled from the published literature [16], with each species categorized as piscivorous, omnivorous, or herbivorous.

### 2.4. Resource Selection Function

To address whether habitat quality or heterospecific flocking opportunities better predicted site use by the solitary Scaly-sided Merganser, we employed a used-available Resource Selection Function (RSF) framework [11], which compared characteristics of used sites versus available but unused sites to quantify the relative importance of habitat attributes and the presence of potential flocking partners. The five ice-free patches represented the available sites, with the two sites where the bird was consistently observed (Sites 2 and 4) designated as “used” (1) and the remaining three sites (Sites 1, 3, 5) as “available” (0). We conducted the RSF analysis at the site level, treating each of the five ice-free patches as an independent sampling unit, to avoid pseudoreplication from repeated observations of the same individual across the three winter seasons. The ‘available’ sites were defined as those ecologically accessible to the individual: (1) they are ice-free water patches within the same river system with no physical barriers preventing access, and (2) they support other waterbird species, indicating suitable foraging conditions. Because only three available sites existed, we generated pseudo-absence points following Northrup et al. [19]. For each of the three available sites, we generated 15 pseudo-absence points by randomly perturbing continuous variables within ±0.1 SD while constraining categorical variables to original levels. Used points were assigned weight = 1, available points weight = 5000, yielding 2 used and 48 available points (3 real + 45 pseudo-absence).

We standardized all continuous predictors (cover score, disturbance score, Common Merganser abundance) to mean = 0 and unit variance, while retaining flow speed as an unstandardized categorical variable. To directly compare the predictive power of habitat attributes versus the presence of potential flocking partners, we developed four candidate models using weighted binomial logistic regression: (1) Null model (intercept only), (2) Habitat-only model (standardized cover score + standardized disturbance score + unstandardized flow speed), (3) Common-only model (standardized Common Merganser abundance), and (4) Full model (all variables combined). We used AICc for model selection [20], calculating ΔAICc and AICc weights for each model. Models with ΔAICc < 4 were considered substantially supported. We performed model averaging across models with ΔAICc < 4 using the zero-method in the MuMIn package (version 1.48.19), and variable importance was calculated as the sum of AICc weights of all models containing that variable.

### 2.5. Small Sample Habitat Association Analysis

Due to the small number of used sites (n = 2), which caused complete separation in logistic regression, we supplemented the RSF analysis with descriptive statistics to examine associations between site use and categorical habitat variables (concealment: high vs. low; disturbance: low vs. high; Common Merganser presence: present vs. absent). We also performed Fisher’s exact test as an exploratory analysis, given its appropriateness for small-sample contingency tables.

### 2.6. Flocking Analysis

We constructed three generalized linear models with a binomial error structure to evaluate predictors of flocking association: genetic distance only, dietary similarity only, and the additive model to test whether phylogenetic relatedness or dietary similarity better predicted flocking. Due to the small number of sympatric species (n = 7), we did not interpret coefficient estimates directly; instead, we used AICc-based model comparison to assess relative support for each predictor [21]. The predicted probability of flocking for each species was derived from the best-supported model. The predicted probability of flocking for each species derived from the best-supported model. All analyses were performed in R version 4.5.3.

## 3. Results

### 3.1. Flocking Behavior and Species Associations

A total of eight waterbird species were recorded across the five survey sites (Table 2). The Scaly-sided Merganser was observed only at sites 2 and 4. The Common Merganser was present at four of the five sites (absent only from Site 3), with peak counts ranging from 10–30 individuals.

**Figure 2:**
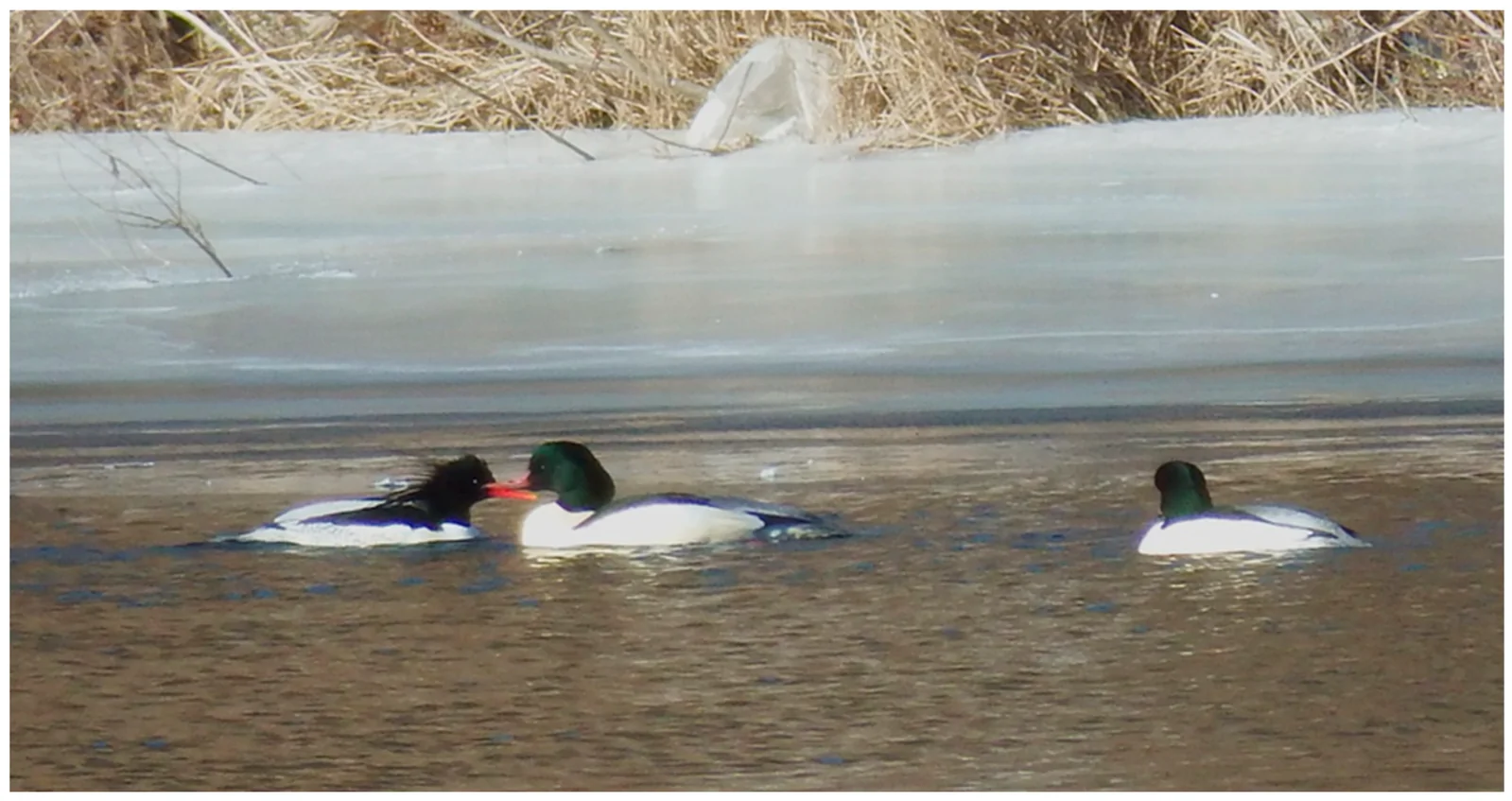
Photograph capturing the focal solitary scaly-sided merganser (left) alongside two common mergansers at Site 4. Photo taken by Guodong Yi.

The solitary male Scaly-sided Merganser was consistently observed in close association with Common Mergansers (Figure 2). At sites 2 and 4, the bird was always found within 5–10 m of at least one Common Merganser and was rarely observed alone. The two species exhibited synchronized behavioral rhythms, and their strong temporal coordination was reflected in aligned activity patterns because they tended to conduct foraging, swimming and resting within the same time windows. Additionally, they showed similar time budgets across all activity types, with foraging being the dominant activity for both species, followed by swimming and resting. A Wilcoxon rank-sum test was used to evaluate interspecific variation in time allocation to the five recorded behaviors (foraging, swimming, resting, preening, playing), with no statistically significant differences identified between species (all *p* > 0.05). Whenever the Common Merganser flock relocated within the ice-free river reach, the focal Scaly-sided Merganser consistently followed. Despite the presence of other waterbird species, the Scaly-sided Merganser was never observed associating with them.

### 3.2. Behavioral Drivers of Site Use and Flocking Association

**Figure 3:**
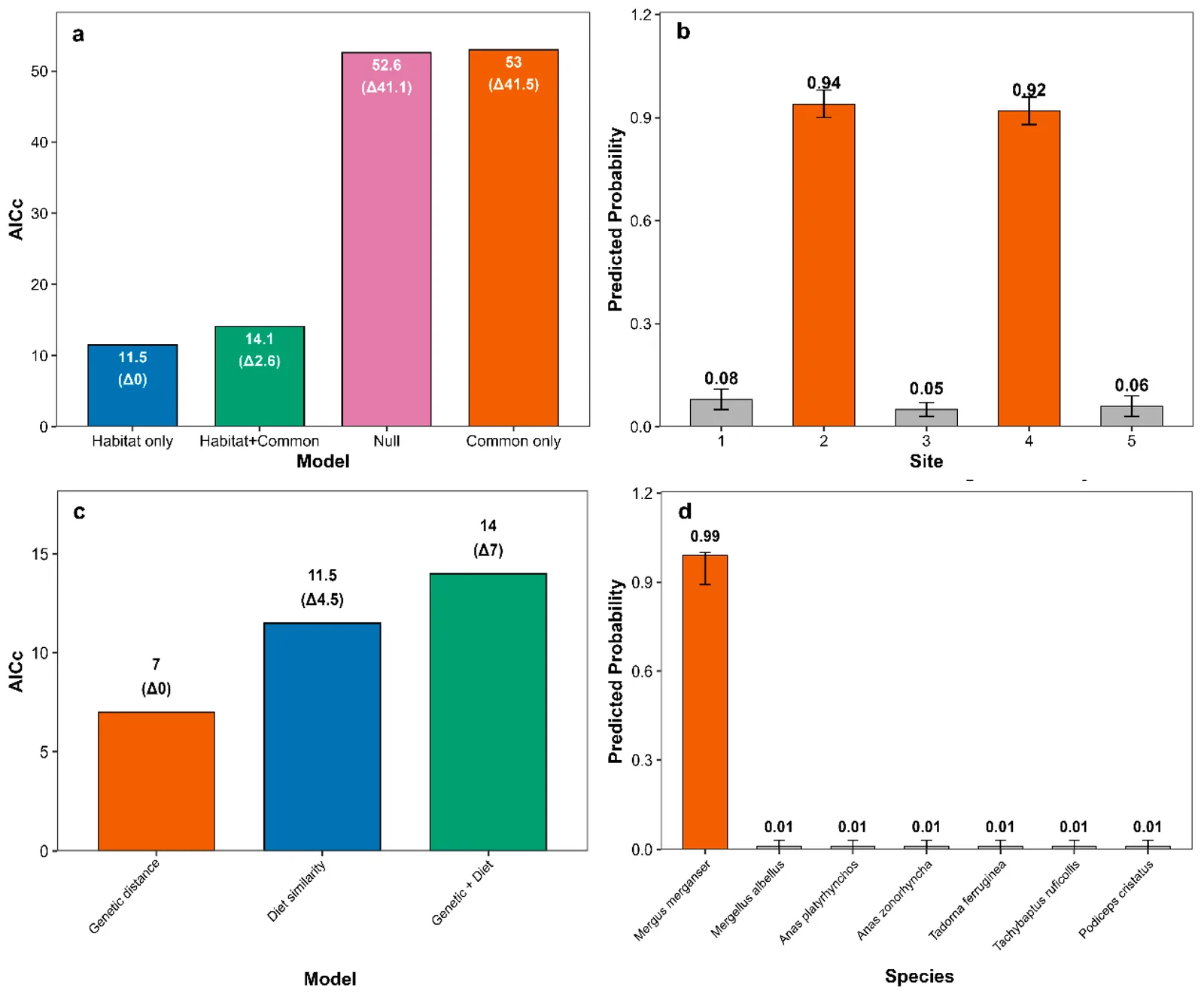
Habitat selection and flocking behavior analyses. (**a**) RSF model comparison showing AICc values for four candidate models: Null, Habitat only, Common only, and Full model. ΔAICc values (difference from the best model) are shown above each bar. (**b**) Predicted probability of site use by the solitary Scaly sided Merganser across the five ice free patches (Sites 1–5). Probabilities were derived from model averaging of top RSF models (ΔAICc < 4). Error bars represent ±1 SE. Bar colors indicate observed use (orange = used, grey = not used). (**c**) Flocking model comparison showing AICc values for three candidate models testing effects of genetic distance and dietary similarity. (**d**) Predicted flocking probability for each sympatric waterbird species from the best supported model (Genetic distance only, AICc = 7.0). Error bars represent 95% confidence intervals. Bar colors indicate observed flocking status (orange = flocking observed, grey = no flocking observed).

The RSF analysis showed that habitat characteristics were the primary determinants of site use for the solitary Scaly-sided Merganser, substantially outweighing the presence of potential flocking partners (Common Mergansers). The habitat-only model achieved the lowest AICc (11.5), followed by the full model (14.1, ΔAICc = 2.6), while the common-only model had a substantially higher AICc (53.0, ΔAICc = 41.5) and received negligible support (Figure 3a). AICc weights were 0.79 for the habitat-only model and 0.21 for the full model.

The predicted probability of site use exceeded 0.9 for Sites 2 and 4, while predicted probabilities were below 0.1 for all other sites (Figure 3b). All used sites (n = 2) were characterized by high concealment and low human disturbance, whereas all unused sites (n = 3) exhibited low concealment and high human disturbance (Table 3). In contrast, Common Merganser presence was similar between used and unused sites (Table 3). Across three winter seasons, the sites we observed were consistently separated by habitat attributes—those used by our focal bird were clearly different from those avoided. This suggests that habitat quality mattered more than the presence of potential flocking partners in determining where this bird spent its winters.

AICc-based model comparison of flocking association revealed that the genetic distance model was best supported (AICc = 7.0, weight = 0.76), while the dietary similarity model received substantially less support (11.5, ΔAICc = 4.5, weight = 0.10; Figure 3c). The predicted probability for the Common Merganser exceeded 0.99, while all other species had probabilities < 0.01 (Figure 3d).

## 4. Discussion

A key question arising from this study is whether this individual’s behaviour represents an atypical response by an anomalous individual, a unique behavioural strategy adopted by a normal individual under environmental stress, or a more general pattern for solitary wintering conspecifics. We consider the individual to be behaviourally normal. Over the three-year tracking period, its daily behavioural repertoire, including foraging, swimming, resting, preening, and playing, did not differ qualitatively from patterns documented in southern wintering flocks. The only notable adjustment was a local temporal synchronisation of activity rhythms with Common Mergansers, reflecting fine-scale behavioural flexibility rather than abnormality [10]. Whether these patterns are representative of the species as a whole, however, cannot be determined from our data due to a lack of conclusive evidence. If the focal individual had been different each year, the behavioural similarity observed over three consecutive winters would offer some evidence for a species-level pattern. Unfortunately, we have no way of confirming whether the three wintering records represent the same male or three different individuals. Given that the Benxi region is not a typical wintering ground for this species, the presence of any Scaly-sided Merganser here is a rare event. It therefore seems highly probable that the same bird returned each winter. Nevertheless, our interpretations remain at the individual level, pending the discovery of additional cases in future surveys to test the generality of our findings.

### 4.1. Habitat Selection Takes Precedence over Flocking Opportunities

Our findings reveal that the solitary wintering Scaly-sided Merganser prioritized habitat quality over social opportunities. Although Common Mergansers occurred at four of the five ice-free patches, the focal individual used only the two most concealed sites (Sites 2 and 4). A clear directional trend was observed for both concealment and disturbance in relation to site use (*p* = 0.10 for each), although statistical significance at α = 0.05 was not reached—most probably reflecting the very limited sample of used sites (n = 2). Critically, this habitat preference was not a transient or opportunistic choice: over three consecutive winter seasons (2019–2022), the focal male consistently occupied only Sites 2 and 4, returning to the same two sheltered, low-disturbance patches each year despite the availability of alternative ice-free sites containing Common Merganser flocks. This three-year consistency, combined with the perfect separation in the habitat data, provides strong observational evidence that the observed site selectivity reflects a genuine, repeated behavioral preference rather than stochastic annual fluctuation. The lack of association between Common Merganser presence and site use (*p* = 1.00) further indicates that the bird did not actively seek areas with high congener densities. A clear example is the contrast between Sites 2 and 5: both had similar flow velocities and hillside adjacency, but the bird used Site 2 exclusively because it was sheltered from roads, whereas Site 5 had ice fishing and road proximity. Thus, fine-scale variation in human disturbance, rather than broad habitat categories, determined site use.

Similar patterns have been observed in other wintering waterfowl. Wintering Scaly-sided Mergansers adjusted diving behavior primarily in response to environmental conditions (with flock size playing a secondary role [8], whereas human disturbance primarily increased their alert time [22]. Migratory waterbird research at Shenzhen Bay also confirmed local human activity acts as the dominant habitat filter. Protected zones lose conservation value once human density and night lighting cross critical thresholds [23]. Our findings extend this general pattern to a solitary wintering individual, demonstrating that habitat quality outweighs social factors not only at the population level but also in individual site selection.

This primacy of habitat selection does not imply that social associations are unimportant. Rather, our findings suggest a two-step logic: habitat first, then social association. The focal individual selected ice-free patches based on concealment and low disturbance; having secured a suitable site, it consistently associated with Common Mergansers within those patches. The RSF results indicate that habitat attributes served as the primary filter for site use, while the repeated field observations of mixed-species flocking at Sites 2 and 4 highlight social associations as a critical secondary component of the bird’s wintering strategy. Thus, habitat selection and flocking are complementary rather than competing processes. These individual-level observations carry clear conservation implications for northern river systems with seasonal full ice cover: protecting small, sheltered, low-human-disturbance ice-free refugia is critical for supporting solitary wintering individuals of this endangered merganser when conspecific flocks are absent.

### 4.2. Phylogenetic Relatedness Drives Selective Flocking

Two classic theories explain mixed-species flocking: improved feeding success and lower predator risk [3]. Newer research adds behavioural plasticity to this framework. Species choose which flocks to join and adjust feeding behaviour to match flock partners [24]. In our study system, ecological facilitation appears more relevant than reduced competition, as the solitary Scaly-sided Merganser gains active benefits (enhanced vigilance and shared foraging information) from associating with Common Mergansers.

First, mixed-species flocks reduce predation risk through the “many-eyes” effect, dilution, and visual confusion [25]. By joining Common Mergansers, the focal individual benefits from collective vigilance and reduced per capita predation risk.

Second, interspecific facilitation encourages solitary birds to join flocks and improve foraging success. Within scarce small ice-free river patches, prey becomes concentrated and intraspecific competition rises; established flocks allow individuals to locate prey far more efficiently—a consistent pattern documented in waterbirds across cold winter periods [26]. Common mergansers are the focal bird’s nearest genetic relative (genetic distance = 0.04365), and both hunt the same prey using nearly identical diving behavior. By grouping, the merganser can share limited feeding habitat. This is consistent with previous observations linking similar foraging habits to mixed-flock formation [27]. Shared fish-eating diet alone could not explain flocking choice. The diet-similarity model had weak statistical support (AICc = 11.5, ΔAICc = 4.5, weight = 0.04). Shared feeding habits were not enough to drive association. Close genetic similarity, which carries matching habitat and behavioural traits, acted as the key driver [28]. Published work confirms related species coordinate anti-predator signals more easily than distantly related taxa, lowering the cost of mixed flocking [27]. Hence, the Common Merganser, as the closest relative, is the most behaviorally compatible flocking partner.

Third, it is important to note that the Smew (*Mergellus albellus*) shares the same piscivorous diet and has a genetic distance of only 0.06384, yet it was never observed associating with the focal individual. Field observations revealed that Smew occurred exclusively at Site 1, a site characterized by open water, high human disturbance, and poor concealment—habitat features that the focal individual actively avoided, suggesting that habitat selectivity can override flocking potential even between close relatives.

In summary, the selective association between the solitary Scaly-sided Merganser and the Common Merganser is likely driven by a combination of antipredator benefits, enhanced foraging efficiency through social information transfer and synchronized diving, and phylogenetic compatibility. The extreme scarcity of ice-free patches forces the solitary individual to share limited foraging grounds, and the Common Merganser—being the closest phylogenetic relative with nearly identical behavior—represents the most compatible flocking partner. In contrast, other piscivorous species such as the smew occupy distinct habitats and rarely encounter the solitary scaly-sided merganser (*Mergus squamatus*). Non-piscivorous waterfowl, including eastern spot-billed duck (*Anas zonorhyncha*), mallard (*Anas platyrhynchos*), and ruddy shelduck (*Tadorna ferruginea*), may disrupt the focal bird’s foraging activity, making heterospecific grouping disadvantageous. While these patterns are only documented for this single male, the consistent selective association with its closest phylogenetic relative provides a testable hypothesis for future surveys of other solitary Scaly-sided Mergansers across northern river basins.

### 4.3. Limitations

This study carries several key limitations that must be acknowledged when interpreting our results. First, all behavioral and habitat data derive from only one adult male Scaly-sided Merganser monitored over three winters. All observed habitat preferences and flocking strategies represent individual-specific behavioral patterns, and these findings cannot be generalized to female conspecifics or the entire species. Despite repeated surveys across all five ice-free patches each winter from 2019 to 2022, we never detected additional solitary Scaly-sided Mergansers within the study basin; this lack of replicate solitary individuals stems from the species’ globally endangered status, extremely low local overwintering population density, and limited number of ice-free river refugia in northern China. Second, the small number of used sites (n = 2) constrained the complexity of our resource selection models, and we relied on pseudo-absence generation to run weighted logistic regressions, which may introduce minor uncertainty in predicted site-use probabilities. Third, Fisher’s exact test results for concealment and disturbance (*p* = 0.10) should be interpreted cautiously; the lack of statistical significance at *p* < 0.05 is likely a consequence of the minimal sample size (two used sites) rather than evidence against biologically meaningful habitat preferences, given the perfect separation observed in the data. Fourth, our field surveys could not confirm whether the focal male associated with consistent groups of Common Mergansers across years or formed random temporary mixed flocks, as we lacked genetic markers to individually identify heterospecific flock mates. Future multi-basin surveys covering broader river stretches are required to locate additional solitary Scaly-sided Mergansers, to verify whether the habitat and flocking patterns documented here are consistent across multiple isolated individuals.

## 5. Conclusions

This three-year case study provides the first documentation of a solitary Scaly-sided Merganser wintering in northern China, revealing individual-level trade-offs between habitat selection and heterospecific flocking. Our findings suggest that, for this particular male under extreme winter conditions, habitat quality may take precedence over social opportunities, and phylogenetic compatibility may drive selective mixed-species associations. Whether these patterns hold for other solitary individuals of this species, or for similar waterbirds in other regions, remains to be tested through broader surveys.

## Figures and Tables

**Table 1:** Habitat characteristics of the five unfrozen water patches surveyed along the Taizi River.

Site	Concealment Level	Human Disturbance	Flow Velocity	Key Features
1	Low (1)	High (3)	Slow	Open, road adjacent, no shelter
2	High (3)	Low (1)	Slow	River bend, hillside, trees, sheltered
3	Low (1)	High (3)	Fast	Bridge, open, turbulent water
4	High (3)	Moderate (2)	Slow	Hillside, distant from road
5	Low (1)	High (3)	Moderate	Reservoir outlet, ice fishing activity

Note: Concealment and disturbance were scored on a 3-point scale (1 = low, 3 = high).

**Table 2:** Peak counts, genetic distances, and diets of waterbird species recorded at the five survey sites (2020–2022).

Species	Peak Counts					Genetic Distance	Diet
	Site 1	Site 2	Site 3	Site 4	Site 5		
Scaly-sided Merganser (*Mergus squamatus*)	0	1	0	1	0	—	Piscivorous
Common Merganser (*Mergus merganser*)	30	15	0	12	20	0.04365	Piscivorous
Smew *(Mergellus albellus*)	6	0	0	0	0	0.06384	Piscivorous
Ruddy Shelduck (*Tadorna ferruginea*)	200	5	10	7	30	0.06892	Omnivorous
Mallard (*Anas platyrhynchos*)	400	10	20	8	25	0.07342	Omnivorous
Eastern Spot-billed Duck (*Anas zonorhyncha*)	250	15	10	0	22	0.07359	Herbivorous
Little Grebe (*Tachybaptus ruficollis*)	50	8	0	0	0	0.17559	Insectivorous
Great Crested Grebe (*Podiceps cristatus*)	6	0	0	0	0	0.17723	Piscivorous

**Table 3:** Association between site use and habitat characteristics.

Variable	Used Sites (n = 2)	Unused Sites (n = 3)	Fisher‘s Exact Test
Concealment (high vs. low)	2 high/0 low	0 high/3 low	*p* = 0.1
Human disturbance (low vs. high)	2 low/0 high	0 low/3 high	*p* = 0.1
Common Merganser presence (present vs. absent)	2 present/0 absent	2 present/1 absent	*p* = 1.00

Note: Fisher’s exact test *p*-values are provided for completeness. However, given the small sample size (n = 2 used sites), these *p*-values have limited statistical power and should be interpreted with caution. The complete separation in concealment and disturbance, combined with the three-year consistency of site occupancy, provides stronger evidence of habitat association than the *p*-values alone suggest.

## Data Availability

The original contributions presented in this study are included in the article. Further inquiries can be directed to the corresponding author.
